# Multi-Functional Chitosan Nanovesicles Loaded with Bioactive Manganese for Potential Wound Healing Applications

**DOI:** 10.3390/molecules28166098

**Published:** 2023-08-17

**Authors:** Edwin Davidson, Jorge Pereira, Giuliana Gan Giannelli, Zachary Murphy, Vasileios Anagnostopoulos, Swadeshmukul Santra

**Affiliations:** 1 Department of Chemistry, University of Central Florida, Orlando, FL 32826, USA; jorge.pereira@ucf.edu (J.P.); zachary.murphy@ucf.edu (Z.M.); vasileios.anagnos@ucf.edu (V.A.); 2 NanoScience Technology Center, University of Central Florida, Orlando, FL 32826, USA; giuliana.giannelli@ucf.edu; 3 Burnett School of Biomedical Sciences, University of Central Florida, Orlando, FL 32826, USA

**Keywords:** chitosan, nanovesicles, wound healing

## Abstract

Chronic skin wound is a chronic illness that possesses a risk of infection and sepsis. In particular, infections associated with antibiotic-resistant bacterial strains are challenging to treat. To combat this challenge, a suitable alternative that is complementary to antibiotics is desired for wound healing. In this work, we report multi-functional nanoscale chitosan vesicles loaded with manganese (Chi-Mn) that has potential to serve as a new tool to augment traditional antibiotic treatment for skin wound healing. Chi-Mn showed antioxidant activity increase over time as well as antimicrobial activity against *E. coli* and *P. aeruginosa* PA01. The modified motility assay that mimicked a skin wound before bacterial colonization showed inhibition of bacterial growth with Chi-Mn treatment at a low area density of 0.04 µg of Mn per cm^2^. Furthermore, this study demonstrated the compatibility of Chi-Mn with a commercial antibiotic showing no loss of antimicrobial potency. In vitro cytotoxicity of Chi-Mn was assessed with macrophages and dermal cell lines (J774A.1 and HDF) elucidating biocompatibility at a wide range (2 ppm–256 ppm). A scratch wound assay involving human dermal fibroblast (HDF) cells was performed to assess any negative effect of Chi-Mn on cell migration. Confocal microscopy study confirmed that Chi-Mn tested at the MIC (16 ppm Mn) has no effect on cell migration with respect to control. Overall, this study demonstrated the potential of Chi-Mn nanovesicles for wound healing applications.

## 1. Introduction

For the past decades, wound care has become a global medical challenge [1,2]. Wounds or skin incisions can happen in our daily life due to a wide variety of reasons, such as medical procedures, cuts, excessive pressure, burns, or underlying medical conditions. Wounds can become a serious and chronic illness in many patients. The impact is significant in healthcare system with an estimated cost of more than USD 28 billion on wound care in 2014 [3]. In recent years, a vast amount of effort has been dedicated to understanding the wound healing processes and developing new therapeutic strategies to provide effective care and reduce health costs [4,5].

Wound healing is comprised of overlapping multi-phased processes (e.g., inflammation, new tissue/proliferation, and maturation/remodeling) [6] involving a myriad of complex biochemical signals and cellular interaction. When these processes are orchestrated in a timely manner, they can achieve optimal restoration of tissue integrity and functionality [7,8]. Nevertheless, the restoration process takes time, which makes wounds vulnerable to bacterial infections and highlights the need to effectively prevent infection to avoid complications, such as sepsis and septic shock [9]. Antibiotic-resistant bacteria are an emerging threat to the wound care of patients. To combat against antibiotic-resistant pathogens, there is a pressing need to develop suitable multi-functional therapeutic tools. Ideally, this tool will possess multiple antimicrobial mechanisms of action. An alternative would be to augment antibiotic therapy by supplementing with biocompatible antimicrobial nanocomposite. These alternatives are expected to provide enhanced protection against infections and mitigation of bacterial resistance incidence [10,11]. 

There is an immense body of literature outlining the negative impact of excessive reactive oxygen species (ROS) during the healing process [12,13,14]. In particular, chronic wounds have prolonged inflammatory responses creating an environment that fosters an increased accumulation of ROS, surpassing the inherent antioxidant capacity of cells [15]. This high level ROS accumulation hinders the normal physiological healing phase. As a result, the progression to the cell proliferation phase is affected negatively, which is needed to complete the healing process of a wound [15,16,17,18]. Therefore, development of an antimicrobial formulation that incorporates antioxidant properties is highly desirable for wound healing applications.

Currently, wound healing strategies involve the use of wound dressings (e.g., hydrogels, scaffolds) [19,20], ointments [21], growth factors [22], RNA [23], and/or antibiotic treatments [24], among others [25]. Clinically, the most frequently prescribed treatments for wound care include wound dressings and antibiotics due to application simplicity, good efficacy profile, and cost-effectiveness [26,27]. Any advanced multi-functional wound care strategy must demonstrate good compatibility with antibiotics. This research addresses the above by introducing a multi-functional wound healing formulation which has potential to be administrated as a wound dressing, a topical therapeutic, and/or in combination with antibiotics.

In this work, we assessed for the first time the potential of nanoscale chitosan loaded with manganese for wound healing applications. Chitosan is a promising material due to its degradability, biocompatibility, and antimicrobial activity [28,29,30]. Furthermore, chitosan is known to provide hemostatic and healing stimulation [31]. Chitosan KA01 is a US FDA approved wound dressing that is made of 100% non-woven chitosan fiber material [32].

Our attention was drawn to manganese since it is economical, abundant, and is an essential micronutrient for many metabolic and enzymatic processes in humans [33]. In particular, the presence of manganese ions has been reported to provide stimulation on the proliferation of keratinocytes and fibroblasts cell lines, by inducing the expression of integrins during the proliferation phase on in vivo studies of cell monolayers [34,35]. Additionally, compared to other transition metals, manganese ions provide higher antioxidant effects in the inhibition of microsomal lipid peroxidation and peroxyl radical quenching [36]. Therefore, in this study, manganese (Mn) was incorporated as a bioactive agent within the chitosan (Chi) vesicles (Chi-Mn) to enhance the wound healing process. It is expected that the Chi-Mn when combined with standard antibiotic treatment would exhibit multifunctional attributes (antimicrobial, antioxidant, and cell proliferation capabilities). 

## 2. Results and Discussion

### 2.1. Characterization of Nanoscale Chitosan Vesicles Loaded with Bioactive Manganese Ions

**Figure 1:**
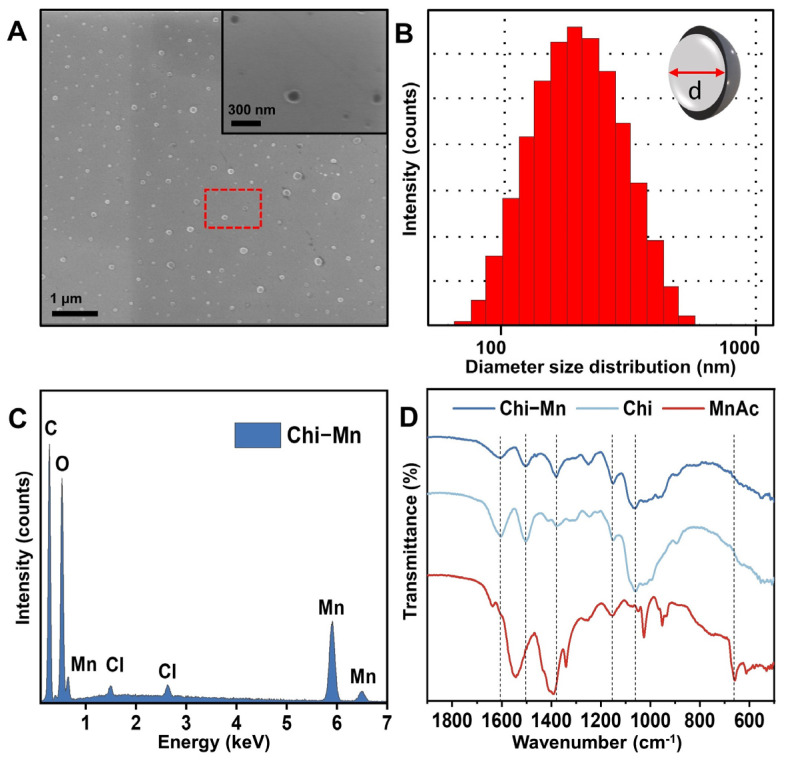
(**A**) SEM images of Chi-Mn. Inset corresponds to a magnified SEM image taken from the region within the red box in (**A**) showing the nanosized vesicles. (**B**) DLS histogram showing the hydrodynamic diameter size distribution of Chi-Mn. (**C**) EDS elemental analysis of Chi-Mn showing the presence of elemental Mn. (**D**) FTIR spectra of Chi, MnAc, and Chi-Mn.

The Chi-Mn was designed to serve as biocompatible nanovesicles carrying antioxidant Mn ions for wound healing applications. One-step facile synthesis of Chi-Mn resulted in the formation of stable colloidal suspension. The SEM images (Figure 1A) revealed the formation of spherical vesicles with a sharp contrast demarcating the outer and inner regions of the vesicles. The average hydrodynamic diameter as measured by the DLS was 150 nm (Figure 1B). The SEM particle size and size distribution corroborated well with the DLS results. The zeta potential value of +24 mV confirmed the positive surface charge of the Chi-Mn nanovesicles. This result is expected as the primary amines groups of chitosan are exposed to the outer surface of the nanovesicles, possibly interacting with the Mn ions. This positive surface charge of chitosan is advantageous for wound healing due to their affinity to negatively charged cells/tissue surface as well as their ability to recruit red blood cells, facilitating hemostasis [37,38].

The UV-Vis spectra of Chi-Mn nanovesicles (Figure S1 of Supplementary Materials) showed the characteristic absorbance peak at 380 nm, which is slightly red shifted when compared to the spectra previously reported by our group [39], suggesting the interaction of chitosan nanovesicle with Mn ions. The formation of the nanovesicles under our modified synthesis protocol is in accordance with Gartner et al. and other groups that evaluated acidic depolymerization of low molecular weight chitosan and the effect of acetate ion induced crosslinking of depolymerized chitosan [40,41].

To demonstrate the presence of Mn within the chitosan vesicles, we conducted EDS mapping of the lyophilized dry powder showing a high signal of Mn (Figure 1C). Furthermore, we recorded and compared the FTIR spectra of the lyophilized powder of Chi, Chi-Mn vesicles, and the manganese (II) acetate hexahydrate control. Table S1 of Supplementary Materials summarizes the major peaks of Chi-Mn, Chi, and MnAc, showing no significant difference between Chi-Mn and Chi spectral signature except for the C=O stretch. The observed Chi-Mn major bands are 1603 cm^−1^ for C=O stretch, 1502 cm^−1^ for N-H bend, 1379 cm^−1^ for C-H bend, 1150 cm^−1^ for C-O stretch, and 1061 cm^−1^ for C-O-H stretch [42]. It is worth noting that Chi-Mn has minor band shifts when compared to chitosan unloaded vesicles in the peaks corresponding to the N-H bend, C-O-H stretch, and C=O stretch. The MnAc characteristic band at 613 cm^−1^ for Mn-O vibration band is not present in the Chi-Mn spectrum [43]. Altogether, these results suggest that the detected Mn in the chitosan vesicles is interacting with the carbonyl (residual acetyl group) and amine groups of chitosan. This finding is similar to what has been previously reported in the literature that Mn ions chelate with chitosan, forming complexes [44,45,46,47]. Guan et al. reported that metal ions (e.g., Mn) could be present partly in solution (free form) as well as remain associated with chitosan surface (metal-ligand complex) without causing flocculation [48,49].

Finally, the ICP-MS quantified the amount of Mn in the purified Chi-Mn sample. The metallic Mn content of Chi-Mn was estimated to be 2138 ± 4 ppm.

### 2.2. Determination of Antioxidant Activity

We conducted a DPPH assay to determine the percentage of free radical scavenging activity, which correlates the higher value of scavenging activity to antioxidant activity. It was found that Chi-Mn radical scavenging activity was approximately 60% over the course of 2 h (Figure S2 of Supplementary Materials). However, to further understand the antioxidant potential of the Chi-Mn, we included appropriate controls of MnAc, Chi, and ascorbic acid (AA). The AA is a well-known antioxidant agent that has strong radical scavenging activities. AA exhibited about 93% scavenging activity (Figure S2) in the first 30 min. This level of activity sustained over 4 h of recorded duration. Alternatively, Chi achieved less than 20% antioxidant activity (Figure S2). The above antioxidant activity results of Chi and Chi-Mn suggest that Mn ion is primarily responsible for exhibiting the antioxidant activity, and therefore it is a bioactive species. MnAc exhibited similar behavior to the Chi-Mn, suggesting no loss of Mn bioactivity despite its association with Chi polymeric matrix. Even though AA exhibited higher antioxidant activity than our Chi-Mn, the Mn loaded nanovesicles demonstrated the advantage of sustained antioxidant activity. This is a desirable property of a wound healing treatment. According to previously reported studies, treatments with slower antioxidant kinetics are beneficial for wound healing applications due to sustained ROS protection [15,50,51,52,53]. The antioxidant activity shown by Chi-Mn was higher than the chitosan nanoparticles reported by Divya et al. [54], but comparable to the activity of previously reported manganese nanoparticles by Zhang et al. [55]. 

### 2.3. Antimicrobial Studies

Recently, Puca et al. reported a microbial isolate analysis of infected wounds and antimicrobial resistance species. In this work, Gram-negative bacteria were detected as the most prevalent species in most patients and *P. aeruginosa* and *E. coli* had the highest occurrence [56]. Additionally, *P. aeruginosa* is designated as a serious threat by the US Center for Disease Control, accounting for about 13% of infections attributed to multi-drug resistant strains [57]. On the other hand, Peralta et al. reported *E. coli* as the most frequent Gram-negative bacteria causing bloodstream infection associated with antibiotic resistance [58]. Overall, both bacteria have a high risk of developing drug resistance by biofilm formation, leading to a severe challenge to surmount in infected wounds [59,60]. For this reason, the antimicrobial activity of Chi-Mn was assessed through MIC and MBC against these common bacterial strains, *E. coli* and *P. aeruginosa*. Similarly, other studies related to wound infection have focused on these two bacterial strains [61,62,63].

In Table 1, we summarized the MIC and MBC for both bacterial strains. The Chi and Chi-Mn treatments exhibited significantly improved (32×) antibacterial efficacy with respect to MIC and MBC values when compared to MnAc (Figure S3 of Supplementary Materials), confirming the interaction of cationic Chi with the negatively charged bacterial cell surface, a contact killing mechanism. In contrast, both Chi and Chi-Mn exhibited reduced antimicrobial activity against *P. aeruginosa* PA01, requiring minimum treatment concentration of 256 µg/mL to prevent bacterial growth. Notably, both Chi and MnAc exhibited distinct MIC and MBC, which suggests that each material might have its own mode of action to inhibit and kill bacteria. 

It is worth noting that our nanoformualtion exhibited significantly higher antimicrobial properties of approximately 10 times more inhibition compared to a previously reported chitosan nanoparticle with manganese ions by Du et al. [64]. In their work, they used an ionic gelation process with Mn ions reporting an MIC and MBC for *E. coli* of 73 and 97 ppm, respectively. Similarly, other reports with chitosan nanoparticles also reported the need of higher concentration of chitosan to achieve the inhibition of *E. coli* [65,66]. Although, some of these chitosan nanoparticles reports [67] have lower MIC against *P. aeruginosa* in the broth dilution method, our nanoformulation exhibited higher potency in a more relevant antimicrobial assay (e.g., motility assay) pertinent to wound healing. 

To combat against the emergence of antibiotic-resistant bacterial strains, a wound dressing platform involving more than one mechanism of action is highly desirable [68]. Wound dressing in combination with oral or topical antibiotic treatments is a standard practice to expedite the wound healing processes and prevent infection [69,70,71]. It is therefore important to evaluate the compatibility of the Chi-Mn with a clinically used antibiotic that is prescribed to mitigate sepsis or severe wound infection. The compatibility of colistin (CO) with Chi-Mn was assessed using a checkerboard assay against *P. aeruginosa* PA01. CO is a re-emerging antibiotic used to combat multi-drug resistant bacterial infection, making it a suitable treatment against resistant *P. aeruginosa* strains [72]. 

**Figure 2:**
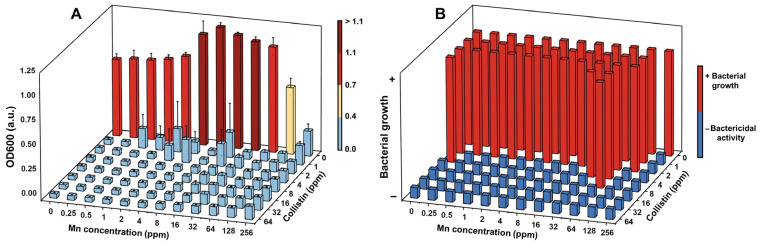
(**A**) Quantification of bacterial growth for *P. aeruginosa* PA01 in response to different concentrations and combinations of Chi-Mn and colistin. The bacterial growth was quantified by measuring OD600 after 24 h of exposure and represented using a heat scale to facilitate visualization. The heat scale values represent the threshold for each level. (**B**) Evaluation of minimum bactericidal concentration based on the in vitro antimicrobial activity shown in (**A**) against *P. aeruginosa* PA01. The error bars represent the standard deviation (*n* = 3).

The results from the checkerboard assay demonstrate that CO inhibits bacterial growth at a MIC concentration as low as 1 mg/mL, while Chi-Mn exhibited an MIC of 256 mg/mL of Mn (Figure 2A). None of the treatment combinations showed antagonistic interaction. These findings demonstrate that Chi-Mn is compatible with CO and could be used against *P. aeruginosa* PA01. Furthermore, we assessed the bactericidal activity (Figure 2B) of the Chi-Mn + CO combination at different concentrations. The results show that Chi-Mn at a concentration of 256 mg/mL Mn is able to lower the minimum bactericidal concentration (MBC) of CO by four times (<1 ppm). This reduction in CO bactericidal concentration is significant, demonstrating the potential benefit of Chi-Mn in improving the antimicrobial potency of CO. 

**Figure 3:**
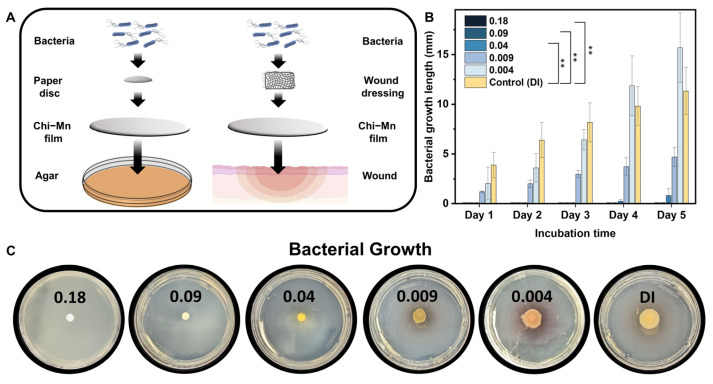
(**A**) Schematic representation of the motility assay mimicking a fresh wound before bacterial exposure. (**B**) Length of *P. aeruginosa* PA01 growth after exposure to different area densities of Chi-Mn in µg of Mn per cm^2^. The error bars represent the standard deviation (*n* = 3). Statistical analysis was performed using one-way ANOVA (** *p* = 0.0021) with a *p* value of 0.05 for Tukey’s post-hoc analysis. (**C**) Optical images captured at day 5 after exposure to different area densities of Chi-Mn in µg of Mn per cm^2^.

To assess the performance of the nanoformulation as a wound dressing, we performed a modified bacterial motility assay by depositing a thin layer of material over agar plates. This assay was designed to mimic a non-infected skin wound before exposure to bacteria (Figure 3A) and has a platform to evaluate the protection of Chi-Mn film barrier against *P*. *aeruginosa* PA01 inoculation. Bacterial growth was evaluated up to day 5 to mimic common wound dressing practices. The results showed that bacterial motility was inhibited at a concentration of 0.04 µg of Mn per cm^2^, while complete bacterial growth inhibition was achieved above 0.09 µg of Mn per cm^2^ (Figure 3B,C). Furthermore, bacterial growth was significantly delayed at a concentration as low as 0.04 µg Mn per cm^2^, showing only 0.82 mm of bacterial growth at day 5. The statistical analysis showed a significant difference in inhibition between the control and the treatment at a concentration as low as 0.04 µg of Mn per cm^2^. It is important to mention that although Chi-Mn exhibited limited in vitro antimicrobial potency against *P*. *aeruginosa* PA01 in the microbroth assay, it can be very effective in preventing bacterial growth at a very low concentration when used as a film barrier. The bacterial motility assay results further confirmed the contact killing potency of Chi-Mn film in comparison to Chi-Mn suspension. Overall, the above results demonstrate the versatility of the Chi-Mn for wound protection as a stand-alone topical therapeutic or as a wound dressing substrate in combination with other antibiotic treatments.

### 2.4. Biocompatibility Studies

Biocompatibility studies involved HDF as model cell line due to their relevance to wound healing. HDF are primary skin cells with critical roles in the complex process of healing, from interactions with keratinocytes cells to synthesis of growth factors [73]. Additionally, we used J774A.1 mouse macrophage to evaluate the effects of Chi-Mn on the immunology with a systemic non-differentiated immune. It should be noted that J774A.1 is widely used in immunology research as a model cell line. Considering that macrophages are also a key cell line during the early stages of healing process providing immune protection and cells organization to accelerate the recovery [74].

**Figure 4:**
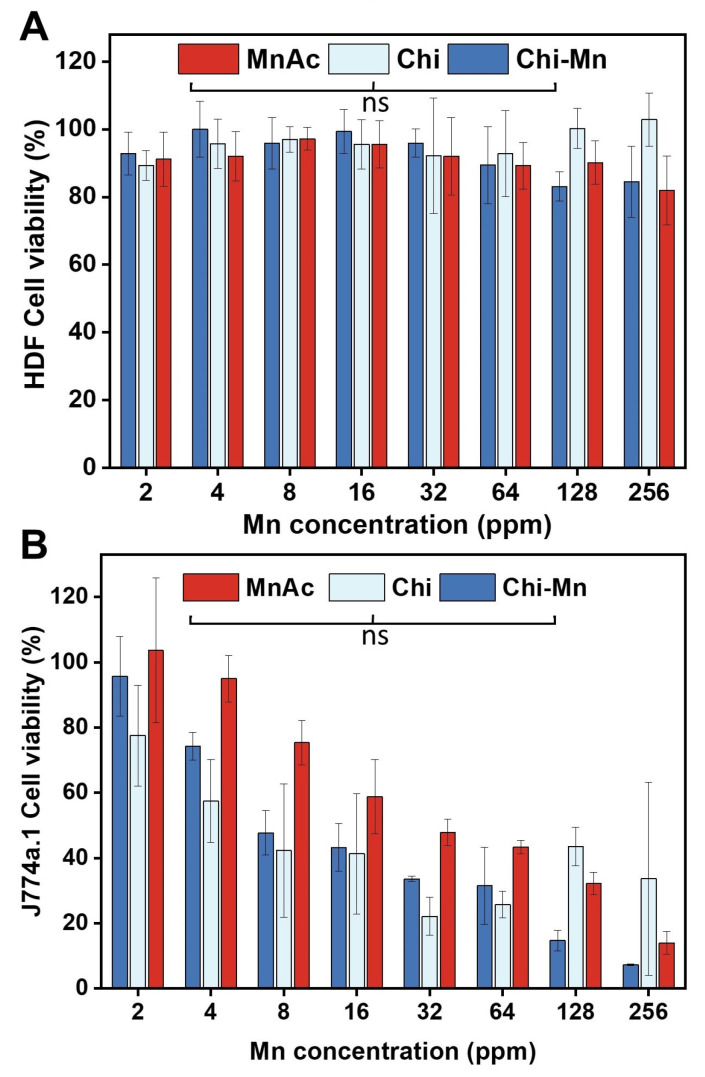
(**A**) Cell viability of human dermal fibroblasts (HDFs) and (**B**) macrophages (J774A.1) upon treatment with Chi-Mn, Chi, and MnAc. Cells were exposed to the respective treatments for 24 h, followed by AlamarBlue reagent for 2 h. Fluorescence was measured at Ex/Em 560/590 nm. Error bars represent standard deviation (*n* = 9). Statistical analysis was performed using Brown–Forsythe and Welch ANOVA test with a *p* value of 0.05 for Dunnett T3 post-hoc analysis.

In vitro biocompatibility was evaluated through an assessment of cell viability of two model cells (HDF and J774A.1) using AlamarBlue cell viability assay that measures the metabolic rates of cells upon exposure to Chi-Mn and corresponding controls (Figure 4). In accordance with ISO 10993-5:2009, percentages of cell viability above 80% are considered non-cytotoxic and lower than 40% are strong cytotoxic [75]. In this study, it was observed that J774A.1 cells experienced severe cytotoxicity at concentration above 8.0 ppm of Mn with Chi, MnAc, and Chi-Mn. This suggests the susceptibility of this cell line to the higher concentration of chitosan and manganese ions, whereas HDF cells do not exhibit cytotoxicity at any of the tested concentrations. As expected, Chi had the highest viability across different concentrations, highlighting why there are many studies and commercial products with chitosan as treatment for wound injuries [37,38]. Comparable HDF viability results were previously reported in other chitosan nanoparticle studies, showing the great biocompatibility of chitosan with this specific cell line [76,77]. 

These results support the duality of Mn ions in a biological system. The excess of Mn can lead to a detrimental impact in cells viability, but under appropriate concentrations it can render beneficial effects. Thus, we demonstrated the biocompatibility of our Chi-Mn when used within the therapeutic window. The statistical analysis by concentration showed no significant difference between Chi-Mn and its individual components, which corroborates the safe combination of MnAc and Chi with no antagonistic effect toward cell viability. More importantly, although the therapeutic window for macrophages is below 8 ppm Mn, our formulation still has bactericidal properties in broth and solid state against *E. coli* and *P. aeruginosa* PA01, respectively. On the other hand, HDF cell line presented a much broader therapeutic window which is a desirable characteristic for our wound healing platform, as we envision Chi-Mn to be used topically in a wound dressing in direct contact with dermal and epidermal cell lines. 

**Figure 5:**
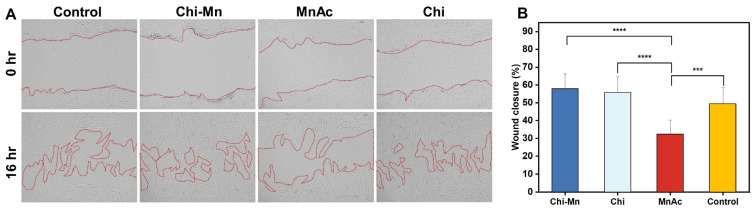
(**A**) Confocal microscopy images of wound scratch assay in HDF cells at time 0 and 16 h after exposure to Chi-Mn, Chi, MnAc, and control (media). Scale bar 100 µm. (**B**) Wound closure percentage represents the mean and error bars represent standard deviation (*n* = 12). Statistical analysis was performed using one-way ANOVA (**** *p* = 0.0001 and *** *p* = 0.0002) with a *p* value of 0.05 for Tukey’s post-hoc analysis.

Furthermore, to obtain a better understanding of the applicability of Chi-Mn for wound healing, we performed a scratch wound assay to assess HDF cell migration in the closure of an artificially generated wound. Regarding wounds, cell’s migration is a pivotal step in the process of wound healing [78]. For these experiments, we utilized open source software ImageJ to analyze the wound area by changing contrast to effectively discriminate the boundary between the cell monolayer and the open wound area. The metallic concentration of Mn was kept constant at 16 ppm on all the experiments involving Mn. The wound closure was assessed at 16 h to evaluate the effects of the treatments on HDF cell migration prior to cell proliferation doubling time. It was observed that the Chi-Mn provided an average wound closure of 57.84%, which was higher than the control (49.30%) and MnAc (32.32%) (Figure 5). Confocal microscopic images showed that the Chi-Mn treatment facilitated cell migrating to the open space of the wound, bridging the gap between both extremities of the cell’s monolayer. Similarly, Chi provided a comparable wound closure of 55.23%, which reiterates the importance of chitosan materials for wound healing. 

Altogether, these results corroborate the biocompatibility of Chi-Mn and its safe potential usage, as a wound dressing or topical therapeutic. This study indeed demonstrated the potential of Chi-Mn nanoformulation as an effective wound dressing providing cell migration and proliferation. Both the combination of proliferation and migration are imperative processes involved in wound closure. At certain concentration range (as demonstrated in the checkerboard assay), the Chi-Mn, in combination with antibiotic treatment could effectively prevent wound infection to facilitate healing.

## 3. Materials and Methods

### 3.1. Synthesis and Characterization

#### 3.1.1. Preparation of Nanoscale Chitosan Vesicles Loaded with Manganese Ions

Water-soluble chitosan vesicles (Chi) were prepared following the protocol of Basumallick et al. with some modifications [39]. Briefly, 150 mg of chitosan low molecular weight (≥75% deacetylated; Sigma Aldrich, St Louis, MO, USA) and 300 mg of manganese (II) acetate tetrahydrate (MnAc) (Acros Organics, Morris Plains, NJ, USA) are dissolved in 30 mL of 1 M hydrochloric acid (Thermo Fisher Scientific, Waltham, MA, USA) to form a semi-fluid solution. Next, the mixture was transferred to a Teflon container (30 mL capacity) and carefully placed within a stainless-steel hydrothermal chamber. Next, the chamber was placed inside an oven (Thermo Scientific, Waltham, MA, USA) pre-heated at 150 °C. After 1.5 h of hydrothermal treatment, the chamber was removed from the oven and cooled down to room temperature. The Chi-Mn product mixture was filtered through a 0.45 µm syringe filter (Thermo Fisher Scientific, Waltham, MA, USA) to remove larger size vesicles. Finally, the product mixture was dialyzed for 24 h in a solution containing equal concentration of manganese acetate to prevent losses of the bioactive, and then the solution pH was raised to approximately 6.0. Hereafter, the resulting purified nanovesicle formulation will be referred to as Chi-Mn. 

#### 3.1.2. Fourier-Transform Infrared Spectroscopy (FTIR)

Prior to FTIR studies, the samples were frozen and lyophilized (FreeZone 4.5 L Freeze Dry System, Labconco, Kansas City, MO, USA) to obtain a dry powder for further analysis. To demonstrate the presence of Mn ions within the chitosan vesicles (Chi), the FTIR spectra of powder Chi-Mn and corresponding controls (MnAc and Chi) were recorded using a Perkin Elmer Spectrum 100 ATR FTIR Spectrometer (PerkinElmer, Waltham, MA, USA).

#### 3.1.3. Scanning Electron Microscopy (SEM)

To evaluate the morphology of Chi-Mn, the sample solution was drop-casted onto a silicon wafer and left to dry overnight in a desiccator with silica gel. The sample was imaged using a Zeiss Nvision 40 (Zeiss, Oberkochen, Germany) with a 5 kV acceleration voltage.

#### 3.1.4. Energy-Dispersive X-ray Spectroscopy (EDS)

The EDS mapping was carried out on samples, which were already prepared for the SEM imaging study. Briefly, SEM images were collected at acceleration voltage of 15 kV with a TM3000 tabletop microscope (Hitachi, Ibaraki, Japan) equipped with an EDS detector followed by the elemental mapping with the Bruker Quantax 70 software version 1.3.

#### 3.1.5. Dynamic Light Scattering (DLS) and Zeta Potential (ζ)

Prior to DLS measurements, the Chi-Mn suspension was filtered through a 0.45 µm syringe filter, dialyzed, and then analyzed with a Zetasizer ZS90 (Malvern Panalytical, Malvern, United Kingdom) to determine the average hydrodynamic size, size distribution, and zeta potential. Particle suspension was prepared using DI water for the DLS and zeta potential measurements.

#### 3.1.6. Ultraviolet-Visible (UV-Vis) Spectroscopy

All the UV-Visible spectra were recorded on an Evolution 220 UV-Vis Spectrophotometer (Thermo Scientific, Waltham, MA, USA) using a 1 cm quartz cuvette.

#### 3.1.7. Inductive Coupled Plasma Mass Spectrometry (ICP-MS)

To determine total Mn content in the nanoformulation, ICP-MS (iCAP RQ, Thermo Scientific, Waltham, MA, USA) was used. Internal standards of scandium (Inorganic Ventures, Christiansburg, VA, USA) and germanium (SPEX CertiPrep, Metuchen, NJ, USA) were used to correct any instrumental drift. A calibration curve of 10–500 ppb Mn was prepared, and samples were diluted to approximately 100 ppb Mn with 2% nitric acid for analysis. Measurements were conducted using kinetic energy discrimination (KED) to account for diatomic interferences.

### 3.2. Determination of Antioxidant Activity

The antioxidant activity of a formulation is commonly assessed through free radical scavenging assays. To determine the free radical scavenging capacity, the 1,1-diphenyl-2-picryl hydrazyl (DPPH, Fisher scientific, Waltham, MA, USA) assay was performed following the reported methods with some modifications [79,80]. Briefly, 190 µL of a 0.25 mg/mL DPPH solution was added to a 96-well plate along with 10 µL of Chi-Mn and corresponding controls (Ascorbic acid, MnAc and Chi) at a concentration of 5 mg/mL. Then, the mixture was left for incubation in the dark from 0.5 to 4 h. The absorbance of the suspension was recorded at 517 nm in different time intervals up to 4 h using a plate reader (Infinite 200Pro Tecan absorbance, Männedorf, Switzerland). The radical scavenging activity percentage was then calculated using the following equation:

$$RSA\;\%=\frac{Abs_{control}-\left(Abs_{treatment}-Abs_{blank}\right)}{Abs_{control}}*100$$

Abs treatment is the absorbance of the treatment, Abs blank is the absorbance of the treatment with no DPPH, and Abs control is the absorbance of DPPH with no treatment. The scavenging activity experiment was conducted in triplicates. 

### 3.3. Antimicrobial and Biocompatibility Studies

#### 3.3.1. Determination of Minimum Inhibitory Concentration (MIC) and Minimum Bactericidal Concentration (MBC)

The antimicrobial properties of Chi-Mn were investigated by calculating MIC and MBC values, elucidating the lowest concentration needed to inhibit bacterial growth and to completely prevent bacterial colony formation, respectively. MIC experiments were performed using the broth microdilution method following the guidelines from Clinical and Laboratory Standards Institute [81]. We tested a concentration range from 2 to 256 µg of Mn/mL of the Chi-Mn formulation and the controls (MnAc and Chi). Both bacterial strains, *E. coli* K-12 (ATCC 29181, Manassas, VA, USA) and *P. aeuroginosa* PA01 (ATCC 15692, Manassas, VA, USA), were grown with nutrient broth and LB Miller broth (Fisher scientific, Waltham, MA, USA), respectively. In all antimicrobial studies, the final bacterial concentration in each well was 5 × 10^5^ CFU/mL. After the addition of the treatments, the plates were covered, sealed, and incubated for 24 h at 37 °C under 150 rpm agitation. A plate reader (Infinite 200Pro Tecan absorbance, Männedorf, Switzerland) was used to measure the absorbance at 600 nm (OD600). To determine the MBC, 100 µL of the bacterial suspensions treated with two concentrations above and two below the MIC value were plated on agar petri dishes. Then, the petri dishes were incubated for 24 h at 37 °C under 150 rpm agitation. All experiments were conducted in triplicates. 

#### 3.3.2. Checkerboard Assay

To assess the effect of Chi-Mn on the antimicrobial activity of colistin (CO), a checkerboard assay was carried out using a published protocol [82]. Initially, 50 µL of Difco LB Miller broth (Fisher scientific, Waltham, MA, USA) was dispensed in all wells of a sterile 96-well plate. Subsequently, 50 µL of Chi-Mn was pipetted into the first column, except for the first row in which 50 µL of 2× Chi-Mn was dispensed. This treatment was then serially diluted until the penultimate column to achieve a final concentration gradient of 256–0.25 mg/mL of Mn. Subsequently, 50 µL of CO solution was added to the first row and serially diluted until the second to last row of the well plate to achieve a final concentration gradient of 64–1 mg/mL of CO. Finally, 50 µL of LB broth and then 100 µL of bacterial suspension at 10^6^ CFU/mL were added to each well. The plates were then sealed with parafilm and incubated for 24 h at 37 °C under 150 rpm agitation. After incubation, the absorbance at 600 nm (OD_600_) was measured with the Infinite 200Pro Tecan plate reader.

To assess the bactericidal activity of the drug combination, 25 µL of the treated bacterial solutions were inoculated into sterile 96-well plates containing 50 µL of LB Miller agar. These plates were inspected for colony growth after 24 h of incubation at 37 °C. All experiments were conducted in triplicates.

#### 3.3.3. Bacterial Motility Assay

The motility of *P. aeruginosa* PA01 was evaluated following the previously reported protocol of Bernal-Mercado et al. with some modifications [83]. First, we deposited Chi-Mn at different concentrations on the surface of Difco LB Miller agar (Fisher scientific, Waltham, MA, USA) petri dishes to form a film. Separately, bacterial suspensions were grown in LB Miller broth for 24 h at 37 °C and 150 rpm. Afterwards, the bacterial suspensions were adjusted to ~10^8^ CFU/mL and then diluted to ~10^6^ CFU/mL before inoculating a paper disc in the center of the petri dishes with 20 µL of the bacteria suspension. The plates were incubated at 37 °C and the length of bacterial growth around the disc was measured daily for 5 days. All tests were conducted in triplicates. The statistical analysis was performed with GraphPad Prism 9.4.1 (GraphPad Software Inc., Boston, MA, USA) using a one-way ANOVA (*p* value 0.05) with Tukey’s post-hoc analysis to determine statistical differences between the treatments.

#### 3.3.4. Determination of Cell Viability

To evaluate the effect of Chi-Mn on cell viability in vitro, we conducted a cytotoxicity assay following the guidelines from ISO 10993-5:2009 [75]. Human dermal fibroblasts (HDF, ATCC PCS-201-012, Manassas, VA, USA) and murine macrophages (J774A.1, ATCC TIB-67, Manassas, VA, USA) were obtained from the American Type Culture Collection (ATCC). Dulbecco’s modified Eagle medium (DMEM, Thermo Scientific, Waltham, MA, USA) with 1% penicillin/streptomycin and 10% heat-inactivated fetal bovine serum (Thermo Scientific, Waltham, MA, USA) was used to culture cells in a T-75 flask under incubation at 37 °C at 5% CO_2_. Both cell types were cultured until 80–90% confluency and then seeded into a 96-well tissue culture microplate at a density of 1 × 10^4^ cells per well. After 24 h of incubation, the old media containing treatments were replaced with a new medium containing 10% AlamarBlue cell viability reagent (Thermo Scientific, Waltham, MA, USA). The cells were then incubated for 2 h at 37 °C at 5% CO_2_, allowing viable cells to metabolize the dye to highly fluorescent product. Using a plate reader (Infinite 200Pro Tecan absorbance, Männedorf, Switzerland), fluorescence intensity was measured at 590 nm upon excitation at 560 nm. The cell viability percentage (CV%) was calculated using the following equation:

$$CV\%=\frac{F_{treatment}}{F_{untreated\;}}*100$$

F treatment corresponds to the fluorescence intensity of the treatment and F untreated is the fluorescence intensity of the untreated control. All experiments were conducted in triplicates. The statistical analysis was performed with GraphPad Prism 9.4.1 (GraphPad Software Inc., Boston, MA, USA) using a Brown–Forsythe and Welch ANOVA (*p* value 0.05) with Dunnett T3 post-hoc analysis to determine statistical differences between the treatments.

#### 3.3.5. Determination of In Vitro Wound Healing Activity

In order to evaluate the wound healing activity of Chi-Mn, we performed a scratch wound assay following the protocol of Martinotti et al. with some modifications [84]. The human dermal fibroblast (HDF) was utilized as the model cell line to mimic a skin wound incision as well as for the determination of the cell migration, an important step in wound healing. HDF was cultured following the same conditions and protocol as described in the cell viability assay (Section 3.3.4). Once 100% confluency was achieved, the cells were seeded to a 24-well tissue culture microplate at a density of 1.3 × 10^5^ cells per well and left for overnight incubation. Then, we proceeded to scrape the cell monolayer in a straight line using a 10 µL pipette tip from west to east and north to south to create a cross. Subsequently, the medium was aspirated from the well and the monolayer was washed with PBS to remove detached cells. Next, Chi-Mn and the controls were added at a concentration of 16 µg of Mn/mL to the cell monolayer. Immediately after addition of the treatment, the first set of images were acquired using a laser confocal microscope (Keyence BZ-X800, Osaka, Japan) in phase contrast mode with a 10× objective. Finally, we followed the method of Suarez-Arnedo to manually measure the wound area coverage using open access ImageJ software version 1.53t [85]. The quantitative data were obtained by measuring the wound area coverage at time point 0 and after 16 h. Then, the wound closure percentage was calculated using the following equation:

$$Wound\;Closure\;\%=\frac{A_{t=0}-A_{t=16}}{A_{t=0}}*100$$

where *A_t_*_= 0_ is the initial wound opening area (in μm^2^) at time 0 and *A_t_*_= 16_ is the wound area after 16 h from the initial scratch. All experiments were conducted in triplicates. The statistical analysis was performed with GraphPad Prism 9.4.1 (GraphPad Software Inc., Boston, MA, USA) using one-way ANOVA (*p* value 0.05) with Tukey’s post-hoc analysis to determine statistical differences between the treatments.

## 4. Conclusions

In the present proof-of-concept study, we successfully demonstrated the usefulness of hydrothermal-based synthesis process (one-step and facile), leading to the formation of multifunctional nanoscale chitosan vesicles incorporated with bioactive Mn ions. This study demonstrated the antimicrobial activity and compatibility of Chi-Mn as well as its potential use in combination with an antibiotic treatment to prevent wound infections. Additionally, the Chi-Mn nanoformulation was biocompatible fostering cell proliferation and cell migration within the safe therapeutic window. This demonstrated the promising functionality of Chi-Mn nanoformulation to protect against infection and promote the wound healing process. The Chi-Mn demonstrated slow antioxidant kinetics providing a temporal increase in the scavenging of free radicals’ activity, which in theory, are expected to provide a prolonged protection against ROS species.

Future studies are required to explore the mode of action of Chi-Mn against clinically relevant bacterial strains and the Mn release kinetics in the tissue level. To the best of our knowledge, this is the first report of hydrothermally synthesized nanoscale chitosan vesicles with manganese acetate, demonstrating multi-functional activities (antioxidant, antimicrobial, and cell proliferation booster), which are important for effective wound healing. We believe that the Chi-Mn has the potential to serve as a wound dressing substrate in a hydrogel or as a topical therapeutic for cutaneous wound injuries, offering a novel treatment alternative.

## Figures and Tables

**Table 1:** MIC and MBC of MnAc, Chi, and Chi-Mn.

	MnAc		Chi		Chi-Mn	
Bacteria	MIC	MBC	MIC	MBC	MIC	MBC
*E. coli* K-12	256	>256	8	8	8	8
*P. aeruginosa* PA01	256	>256	256	256	256	>256

## Data Availability

The data presented in this study are available on request from the corresponding author.

## Supplementary Materials

The following supporting information can be downloaded at: https://www.mdpi.com/1420-3049/28/16/6098/s1. Table S1: FTIR spectral data comparison of MnAc, Chi, and Chi-Mn; Figure S1: UV-Visible spectra of Chi-Mn, Chi, and MnAc; Figure S2: Radical scavenging activity percentage of Mn-Chi in comparison with Ascorbic Acid, MnAc, Chi, and Chi-Mn. Error bars represent standard deviations (*n* = 3); Figure S3: In vitro antibacterial activity of Chi-Mn, Chi, and MnAc against E. coli K-12 and P. aeruginosa PA01. Error bars represent standard deviations (*n* = 9).
