# Supplementary material for: Multi-Functional Chitosan Nanovesicles Loaded with Bioactive Manganese for Potential Wound Healing Applications
Source: Molecules. 2023 Aug 17;28(16):6098. doi: 10.3390/molecules28166098 (PMC10459768; doi:10.3390/molecules28166098)
Supplement: Supplementary file 1 [file molecules-28-06098-s001.zip › molecules-2531073-supplementary.pdf]

Supplementary Information

Multi-Functional Chitosan Nanovesicles Loaded with Bioactive Manganese for Potential Wound Healing Applications

Edwin Davidson, [1,2]* Jorge Pereira, [1,2] Giuliana Gan Giannelli, [2,3] Zachary Murphy, [1] Vasileios Anagnostopoulos, [1] and Swadeshmukul Santra [1,2,3,]*

[1]Department of Chemistry, University of Central Florida, Orlando, FL, 32826, USA.
[2]NanoScience Technology Center, University of Central Florida, Orlando, FL, 32826, USA.
[3]Burnett School of Biomedical Sciences, University of Central Florida, Orlando, FL, 32826, USA.

**Corresponding authors e-mails: edwin.davidsonbarahona@ucf.edu and Swadeshmukul.santra@ucf.edu*

Table of Contents

Table S1. FTIR spectral data comparison of MnAc, Chi, and Chi-Mn. 2

Figure S1. UV-Visible spectra of Chi-Mn, Chi, and MnAc. 3
Figure S2. Radical scavenging activity percentage of Mn-Chi in comparison with Ascorbic Acid, MnAc, Chi, and Chi-Mn. Error bars represent standard deviations (n=3)........ 4
Figure S3. In vitro antibacterial activity of Chi-Mn, Chi, and MnAc against E. coli K-12 and P. aeruginosa PA01. Error bars represent standard deviations (n=9). 5

Table S1. FTIR spectral data of MnAc, Chi, and Chi-Mn.

Literature characteristic absorption band range[a]	MnAc (cm^{-1})	Chi (cm^{-1})	Chi-Mn (cm^{-1})	Assignment of absorption bands
**1690 – 1760**	-	1608	1603	C=O stretch
**1540 – 1660**	-	1503	1502	N-H bend
**1650 – 1360**	1543, 1390	-	-	CO_2 asymmetric and symmetric stretches
**1350 – 1480**	-	1379	1379	C-H bend
**1050 – 1250**	1153	1150	1150	C-O stretch
**1020 – 1200**	1025	1064	1061	C-O-H stretch
**580 – 690[b]**	613, 659	-	-	Mn-O stretch and Mn-O=C bend

[a]Peak ranges were obtained from reference [1] and open access Sigma-Aldrich website.
[b]Peak range was obtained from reference [2].

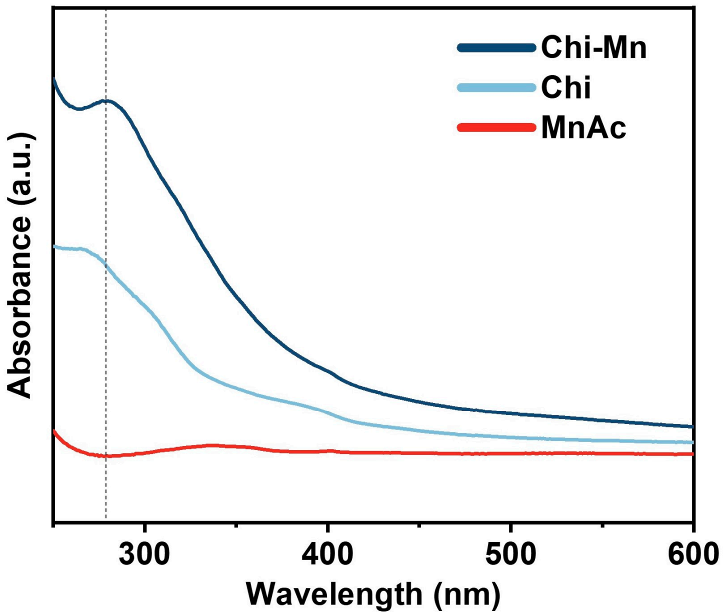

Figure S1. UV-Visible spectra of Chi-Mn, Chi, and MnAc.

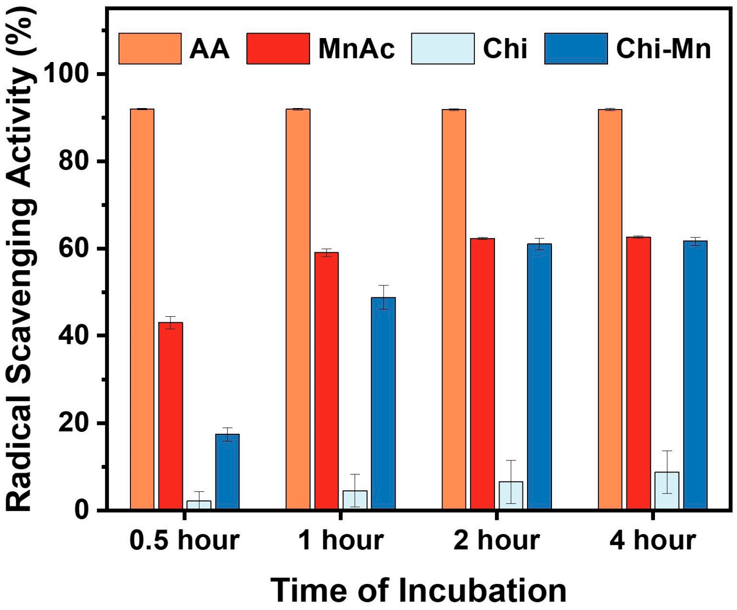

Figure S2. Radical scavenging activity percentage of Chi-Mn in comparison with Ascorbic Acid, MnAc, and Chi. Error bars represent standard deviations (n=3).

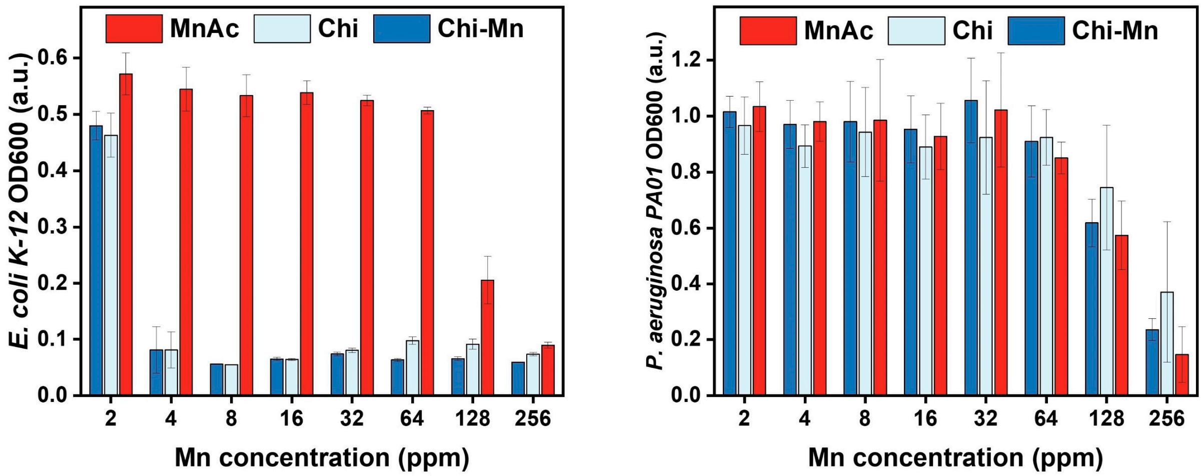

Figure S3. In vitro antibacterial activity of Chi-Mn, Chi, and MnAc against *E. coli* and *P. aeruginosa* PA01. Error bars represent standard deviations (n=9).

References:

1. Larkin, P.J. Chapter 6—IR and Raman Spectra–Structure Correlations: Characteristic Group Frequencies. In *Infrared and Raman Spectroscopy*, 2nd ed.; Larkin, P.J., Ed.; Elsevier: Amsterdam, The Netherlands, 2018; p. 85–134.
2. Cakic, S. Spectroscopic characteristics of highly selective manganese catalysis in acqueous polyurethane systems. *Sensors* **2006**, *6*, 1708–1720.
